# 

**Published:** 1840

**Authors:** 


					THE
AM?IEE(DAM roraMAIL
OF*
DENTAL SCIENCE,
FOR
?Mr?ws3J (S saiFffiaffiiBsms,
Vol I.?No. VII & VIII.
EDITED BY
CHAP IN A. HARRIS, M. D.
Baltimore.
ELEAZAR PARMLY, M. D
New-York.
publishing committee,
E< PARMLY, | E. BAKER,
Si BROWN.
sraw sr#jaTE9
GEORGEADLAKL), 168 BROADWAY,
ARMSTRONG & BERRY.
PEICE?THREE DOLLARS PBS ANNDM.
J. C. Kelley, Printer.

				

